# Dietary multi-enzyme complex improves *In Vitro* nutrient digestibility and hind gut microbial fermentation of pigs

**DOI:** 10.1371/journal.pone.0217459

**Published:** 2019-05-28

**Authors:** Neeraja Recharla, Duwan Kim, Sivasubramanian Ramani, Minho Song, Juncheol Park, Balamuralikrishnan Balasubramanian, Pradeep Puligundla, Sungkwon Park

**Affiliations:** 1 Department of Food Science and Biotechnology, Sejong University, Seoul, Korea; 2 National Institute of Animal Science, Swine division, RDA, Sunghwan, Korea; 3 Division of Animal and Dairy Science, Chungnam National University, Daejeon, Korea; 4 Department of Food Science & Biotechnology, Gachon University, Seongnam, Korea; University of Illinois, UNITED STATES

## Abstract

This study was conducted in two stages to investigate the potential of multi-enzyme supplementation on the nutrient digestibility, growth performance, and gut microbial composition of pigs. In stage 1, effects of multi-enzyme complex (xylanase, α-amylase, β-glucanase, and protease) supplementation on the ileal and total tract dry matter (DM) digestibility of feed-stuffs were investigated with *in vitro* two-stage and three-stage enzyme incubation methods. A wide range of feed ingredients, namely, corn meal, wheat meal, soybean meal, fish meal, Oriental herbal extract, Italian rye-grass (IRG) and peanut hull were used as substrates. Supplementation of the multi-enzyme complex increased (*P* < 0.05) the digestibility of the Oriental herbal extract and corn meal. In stage 2, *in vivo* animal studies were performed to further investigate the effects of the dietary multi-enzyme complex on the nutrient utilization, growth performance, and fecal microbial composition of pigs. A total of 36 weaned pigs were fed corn- and soybean meal-based diets without (control) and with the multi-enzyme complex (treatment) for 6 weeks. Fecal samples were collected from 12 pigs to analyze the microbial communities by using DNA sequencing and bioinformatics tools. Multi-enzyme supplementation had no effect on apparent digestibility of nutrients and growth performance of pigs compared to control. Taxonomic analysis of the fecal samples indicated that the bacteria in both control and treatment samples predominantly belonged to *Firmicutes* and *Bacteroidetes*. In addition, the proportion of the phylum *Firmicutes* was slightly higher in the treatment group. At the genus level, the abundance of *Treponema* and *Barnesiella* increased in the treatment group; whereas the numbers ofthe genera including *Prevotella*, *Butyricicoccus*, *Ruminococcus* and *Succinivibrio* decreased in the treatment group. These results suggest that multi-enzyme supplementation with basal diets have the potential to improve nutrient digestibility and modify microbial communities in the hind-gut of pigs.

## Introduction

Many plant-based feed ingredients used in swine diets, especially cereal grains, contain large quantities of non-starch polysaccharides (NSPs) with anti-nutritional factors (ANFs). NSPs are poorly used by pigs because they lack specific endogenous enzymes for their breakdown, and, consequently, NSPs are fermented and used by pig intestinal microbes [1]. The major NSPs of the plant cell wall are composed of cellulose (linear β-glucan chains), hemicellulose or non-cellulosic polymers (arabinoxylans, mixed-linked β-glucans, mannans, galactans, and xyloglucan) and pectic polysaccharides (polygalacturonic acids) [2]. Elevated levels of NSPs in swine diets have adverse effects on nutrient digestibility and absorption rate. Especially, soluble NSPs increase the viscosity of digesta and alter the intestinal transit time. These effects could, in turn, lead to changes in the physiology and ecosystem of the gut [2,3]. The addition of exogenous NSP- degrading enzymes can improve nutrient availability in swine diets by breaking down the nutrient encapsulating cell wall as well as ameliorating viscosity problems associated with certain NSPs, particularly arabinoxylans and β-glucans [4,5].

Exogenous enzymes are being successfully used in poultry diets to reduce the negative effects of NSPs in cereals such as barley, wheat, and rye [6]. Recently, there has been considerable interest in the use of exogenous enzymes in the swine industry to address the anti-nutritive effects of NSPs on animal performance. A previous study has shown that supplemental exogenous digestive enzymes may improve average daily gain (ADG), feed conversion ratio (FCR), and digestibility of dry matter (DM) in pigs [7]. Thus, supplementation of swine diets with exogenous enzymes has gained popularity, and this approach has the potential to improve the nutrient utilization of high-fiber diets.

Different types of exogenous feed enzymes, such as proteases, carbohydrases, phytases, and xylanases are commercially used in livestock feeds [8]. The inclusion of proteases as feed additives enhances the protein digestion and can increase the growth performance of adult pigs [9, 10]. Proteases also stimulate gut development, maturation, and health in weaning and weaned piglets in the early stages by degrading protein-bound complexes to release other nutrients along with protein [10, 11, 12]. Carbohydrases are enzymes that catalyze the breakdown of complex carbohydrates into oligosaccharides, disaccharides and monosaccharides. Carbohydrases are classified into starch-degradable and NSP-degradable enzymes [13]. The starch-degrading enzymes are not often used in animal feed (except in creep feed) because animals are able to synthesize them endogenously. However, for efficient feed-nutrient utilization, certain enzymes may be exogenously supplemented as feed additives to target NSP hydrolysis in the livestock production industry. Such accessory enzymes include xylanase, mannanase, and glucanase [14, 15]. These enzymes hydrolyze plant cell-wall components such as xylan, mannan, and beta-glucan and assist in the release of nutritional constituents, such as protein, starch, lipids and other minerals, that are trapped within the cell-wall matrix [15, 16, 17]. Upon hydrolysis of NSPs and availability of the entrapped nutrients, the resultant products are readily accessible for the intestinal microflora, which can have multiple beneficial effects on animal gut health and whole animal [18]. Such health-promoting microorganisms enhance gut physiology, for example, reduction of relative weight of organs in the digestive system and increased villus height [18, 19].

Many studies have reported that multi-enzyme supplementation had more positive effects on feed utilization and animal performance when pigs were fed with mixed grain-based diets because of the synergistic interaction between enzymes [20, 21]. The potential of a multi-enzyme preparation for the hydrolysis of different feed-stuffs is principally determined by the digestibility rate. This rate can be measured directly in *in vivo* animal models, but it is practically difficult because of the high number of samples and ethical objection to animal experimentation. In addition, *in vivo* methods are time consuming and expensive procedures. In contrast, *in vitro* incubation techniques that mimic *in vivo* digestion can be considered as rapid methods for the prediction of *in vivo* digestibility values [22, 23]. *In vitro* techniques are relatively less expensive, simpler, and rapid when compared with animal experiments [24]. Therefore, prior to introducing novel exogenous enzymes in swine diets, *in vitro* digestibility methods can be used to determine the efficacy of the exogenous enzymes.

The objective of the present study was to screen swine feed ingredients suitable for hydrolysis by multi-enzyme complex with *in vitro* digestibility methods and to evaluate the effects of enzyme supplementation of corn-soybean meal-based diets on the nutrient digestibility, growth performance, and gut microbial composition of pigs.

## Materials and methods

### 2.1. *In vitro* study

#### 2.1.1. Feed ingredients and enzymes

Seven samples of ground feed ingredients (soybean meal, corn meal, wheat meal, fish meal, Oriental herbal extract, pea nut hull, and Italian rye grass) were obtained and sieved with a 0.85 mm sieve and stored in air-tight containers until digestibility analysis. A commercial exogenous enzyme complex was provided by Feed Best Inc. (Seoul, Korea). The complex contained a mixture of β-pentosanase (xylanases; 6000 EPU/g) and synergetic enzymes, namely, β-glucanase, α-amylase and protease (32,000, 17,600, and 142 EU/g, respectively). The test samples were divided into two groups, feed samples without enzymes that served as the controls, and feed samples with 0.1% multi-enzyme (treatment group).

#### 2.1.2. Experiment procedures: *In vitro* ileal digestibility method

To predict the *in vitro* ileal digestibility (IVID) of the feed-stuffs for pigs, we used the method described by Boisen and Fernandez [23]; it consisted of two-step enzymatic incubations under different pH conditions that simulated digestion in the stomach and small intestine. The experiments were performed with three replicates.

According to the protocol described by Boisen and Fernandez [23], in step one, 1 g of the ground feed sample to an accuracy of ± 0.1 mg (1 g ± 0.1 mg) was placed in a conical flask, 25 mL of 0.1 M phosphate buffer at pH 6 (sodium phosphate buffer prepared with monosodium phosphate and its conjugate base, disodium phosphate) and 10 mL of 0.2 M HCl were added in the flask, and the pH was adjusted to 2 with 1 M HCl or NaOH solution. Then 1 mL of freshly prepared pepsin solution (10 mg/mL; ≥ 250 U/mg solid, P7000, pepsin from porcine gastric mucosa; Sigma-Aldrich, St. Louis, MO, USA) and 0.5 mL of chloramphenicol solution (to prevent bacterial contamination) were added to the mixture. The flasks were incubated in a shaking water bath (shaking speed, 80 rpm) at 39 °C for 6 h. In the second step (after 6 h of incubation), 10 mL of phosphate buffer (0.2 M, pH 6.8) and 5 mL of 0.6 M NaOH solution were added to the mixture in the flasks. Then, pH was adjusted to 6.8 with 1 M HCl or NaOH, and 1 mL of freshly prepared pancreatin solution (50 mg/mL; 4 × USP, P1750, pancreatin from porcine pancreas; Sigma-Aldrich) was added. After enzyme addition, the test flasks were incubated in a shaking water bath at 39 °C for 18 h.

After the incubation, all the samples were placed in an ice bath to stop enzyme action until sample filtration. The undigested residues were filtered using dried and pre-weighed glass filter crucibles containing 500 mg of Celite filter aid (Sigma-Aldrich) with vacuum pump support. All undigested samples were transferred to the filtration crucible by rinsing the flasks with distilled water, and the residue was further washed with about 10 mL of 95% ethanol and 99.5% acetone to remove the lipid content. After filtration, the undigested residue in the crucibles was dried at 101 °C overnight. Then, the residue was weighed and cooled for 1 h in a desiccator. The *in vitro* digestibility of DM was calculated from the difference between DM in the sample and undigested residue after correction for DM in the blank.

**2.1.2a: *In vitro* total tract digestibility method**. *In vitro* total tract digestibility (IVTTD) was measured using a three-step multi-enzymatic incubation method, which consists of gastric (pepsin added), small intestine (pancreatin added), and large intestine (viscozyme added)-simulated digestive phases according to Boisen and Fernandez [23]. The experiments were performed with three replicates.

**Fig 1:**
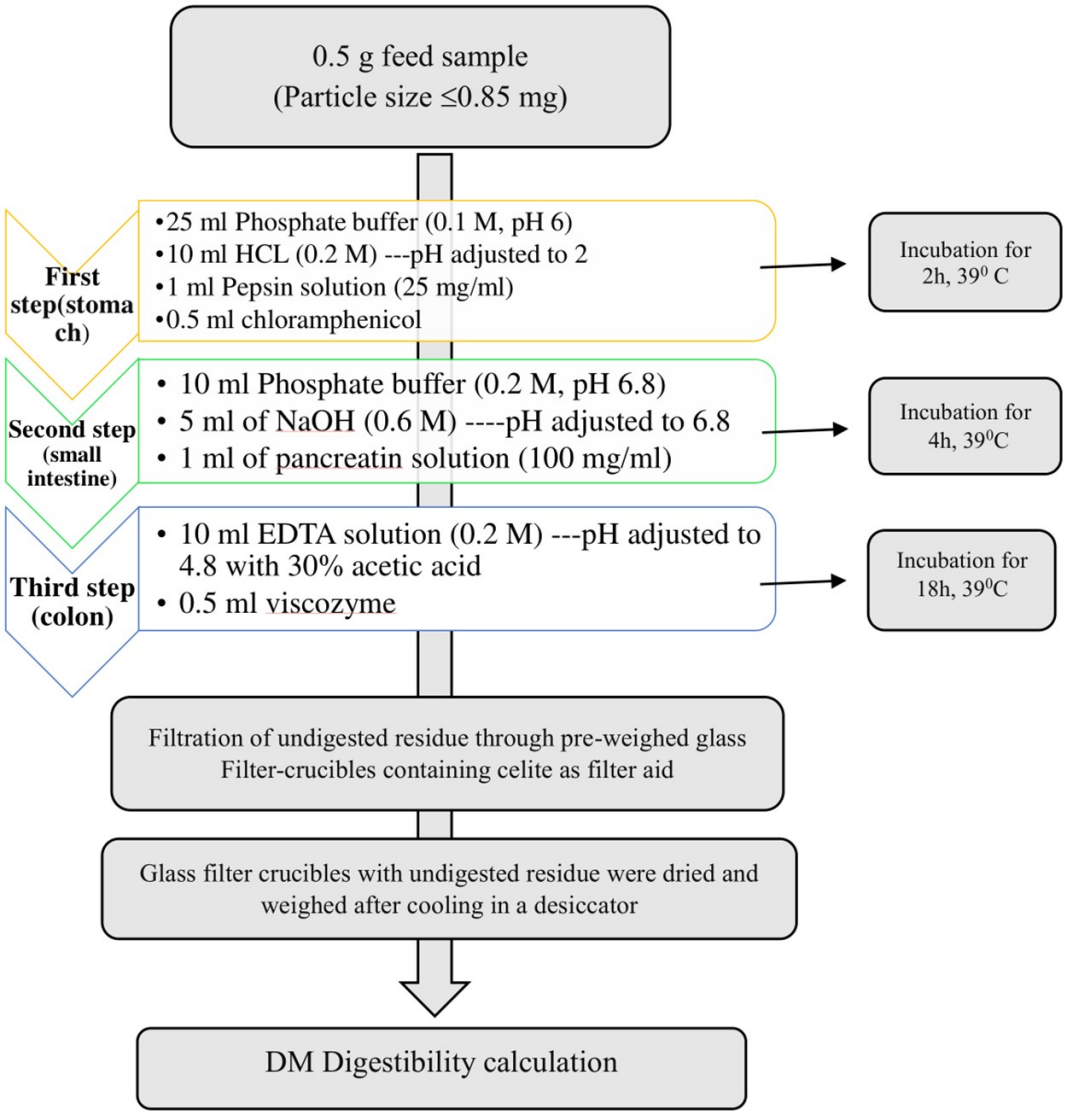
Schematic representation of IVTTD.

The ground feed sample (0.5 g, particle size ≤0.85 mm, accuracy of 0.001 g) was placed in a 100 mL conical flask, and 25 mL of 0.1 M phosphate buffer (pH 6) was added to the flask, pH of the solution was reduced with 10 mL of 0.2 M HCl and the pH adjusted to 2.0 ± 0.1 with 1 M HCl or 1 M NaOH. Then, 1 mL of pepsin solution, which was prepared by adding 25 mg of pepsin per mL of distilled water (pepsin from porcine gastric mucosa, P 7000, ≥ 250 U/mg solid; Sigma-Aldrich) was added. To prevent microbial contamination, 0.5 mL chloramphenicol solution (0.5 g of chloramphenicol in 100 mL of ethanol) was added. The flasks were closed with silicon stoppers and placed in a water bath with mild agitation (50 rpm) at 39 °C for 2 h ± 5 min (first step). After the first incubation, 10 mL of 0.2 M phosphate buffer (pH 6.8) and 5 mL of 0.6 M NaOH were added to the flasks, and the pH was adjusted to 6.8 ± 0.1 with 1 M HCl or 1 M NaOH. Then, 1 mL of pancreatin solution (Sigma-Aldrich) containing 100 mg of pancreatin per 1 mL of distilled water was added to the mixture. The flasks were closed with silicon stoppers and incubated in a shaking water bath with mild agitation (50 rpm) at 39 °C for 4 h ± 5 min (second step). After the incubation, 10 mL of 0.2 M EDTA solution was added to the mixture, the pH was adjusted to 4.8 ± 0.1 with 30% acetic acid solution, and 0.5 mL of Viscozyme L (V-2010; Sigma-Aldrich) was added to each flask; the flasks were incubated in a shaking water bath for 18 h with agitation at 39 °C (third step). Then, the flasks were placed in ice water to stop enzymatic reaction. The schematic representation of the IVTTD method is shown in Fig 1.

IVID and IVTTD of DM (%) were calculated using the following equation;

IVIDorIVTTDofDM%=[SampleDM-(ResidueDMI-BlankDM)]/SampleDM×100

DM = Dry matter

### 2.2. In vivo study

#### 2.2.1. Pigs and housing

A total of 36 weaned pigs [Duroc × (Landrace × Yorkshire) with an initial average body weight (BW) of 6.02 ± 0.32 kg were used for this study. The experimental protocol for this study was reviewed and approved by the Animal Care and Use Committee of Chungnam National University (Approval# CNU-00611). The pigs were randomly assigned to two different dietary treatments, either control or treatment, with three pigs per pen. Each pen was equipped with a feeder and waterer in an environmentally controlled room. The pigs had free access to water and feed.

#### 2.2.2. Experimental diets

The diets were based on corn and soybean meal and formulated to meet required amounts of vitamins and minerals for pigs [25]. The composition of the experimental diets is listed in Table 1. The diets were fed without (control) and with the multi-enzyme supplement (treatment). Treatment diet was the control diet supplemented with 0.1% (w/w) multi-enzyme complex including xylanase, α-amylase, β-glucanase, and protease (6000 EPU/g, 17,600, 32,000, and 142 EU/g, respectively). The pigs were fed for 6 weeks according to a two-phase feeding program: weeks 1 to 3 (phase 1, 21 days) and weeks 4 to 6 (phase 2, 21 days). During the experimental period, two dietary compositions (for phases 1 & 2) were formulated to meet the nutrient requirements based on age. The diets did not include spray-dried plasma, antibiotics, or zinc oxide to avoid their antibacterial or physiological effects.

**Table 1:** Composition of experimental diets fed for weaned pigs.

Ingredient (%)	Phase 1^x^		Phase 2^y^	
	Control	Treatment	Control	Treatment
Corn	31.57	31.57	51.56	51.56
Soybean meal, 44%	18.00	18.00	26.56	26.56
Soy protein concentrate	16.96	16.96	8.00	8.00
Dried whey	24.00	24.00	10.00	10.00
Lactose	4.00	4.00	-	-
Soybean oil	3.00	3.00	1.35	1.35
Limestone	1.00	1.00	1.00	1.00
Multi-enzyme supplement^a^	-	+	-	+
Monocalcium phosphate	0.90	0.90	0.90	0.90
Vitamin pre-mix^b^	0.20	0.20	0.20	0.20
Mineral pre-mix	0.20	0.20	0.20	0.20
L-lysine-HCl	0.08	0.08	0.17	0.17
DL-methionine	0.09	0.09	0.07	0.07
Total	100	100	100	100
**Calculated energy and nutrient content**				
ME, Mcal/kg	3.53	3.53	3.42	3.42
CP, %	24.49	24.49	22.51	22.51
Calcium, %	0.81	0.81	0.73	0.73
Phosphorus, %	0.69	0.69	0.63	0.63
Lysine, %	1.54	1.54	1.41	1.41

Phase 1^x^ = week 1 to 3 (21 days), phase 2^y^ = week 4 to 6 (21 days).

Multi-enzyme supplement^a^ = 1 kg multi-enzyme mixture was mixed per 1 ton of control diets. The multi-enzyme contained xylanase, α-amylase, β-glucanase, and protease.

Vitamin pre-mix^b^ = Provided per kilogram of diet: vitamin A, 12,000 IU; vitamin D_3_, 2,500 IU; vitamin E, 30 IU; vitamin K3, 3 mg; D-pantothenic acid, 15 mg; nicotinic acid, 40 mg; choline, 400 mg; and vitamin B_12_, 12 μg.

ME = Metabolizable energy; Mcal/kg = megacalories per kilogram

CP = Crude protein

#### 2.2.3. Performance monitoring and sample collection for digestibility analysis

The pigs were individually weighed on day 1, 21, and 42 of the experiment and the amount of feed supplied per pen and remaining feed were recorded at the beginning and end of the experiment for each phase. These values were used to measure ADG, average daily feed intake (ADFI), and feed efficiency (gain to feed ratio, G:F).

Diet samples were also collected from each batch of the manufactured feed and stored at -20 °C until analyses. To determine the apparent digestibility of nutrients, 0.25% chromic oxide (as the marker) was added to the diets during days 36 to 42 of the experiment [26]. Fecal samples were collected for 3 days after the 4-day adjustment period for apparent total tract digestibility. The collected fecal samples were pooled and stored at -20 °C until analyses. Two pigs per pen were euthanized using CO_2_ after injection of zoletil and exsanguinated at the end of the experiment. Digests samples from the ileum were collected and stored at -20 °C until analyses.

#### 2.2.4. Analytical methods

The diet, feces, and digesta samples were dried in a force-air drying oven at 60 °C and ground through a cyclone mill (Foss Tecator Sycltec 1093, HillerØd, Denmark) before analysis. The diet, feces, and digesta samples were analyzed for DM (method 930.15) and nitrogen (method 988.05) according to the AOAC methods [27]. Gross energy was measured using a bomb calorimeter (Parr 1281 Bomb Calorimeter: Parr Instrument Co., Moline, IL, USA), and chromium content was determined using an absorption spectrophotometer (Hitachi Z-5000 Absorption Spectrophotometer; Hitachi High-Technologies Co., Tokyo, Japan), according to Williams *et al*. [26]. Apparent ileal and total tract digestibility of DM, CP, and energy were calculated using the index method [28].

#### 2.2.5. Microbial community analysis

To analyze the bacterial diversity in the pig feces, fecal samples were collected from the two groups of pigs (6 pigs per group). The collected fecal samples were immediately stored at -80 °C for further analyses.

**2.2.5a: DNA isolation and PCR amplification**. The DNA was extracted from the fecal samples by using the PowerSoil DNA Isolation Kit (Cat. No. 12888, MO BIO) according to the manufacturer’s protocol. Each sequenced sample was prepared according to the Illumina 16S Metagenomic Sequencing Library protocols. The DNA quantity and quality were measured using PicoGreen (Invitrogen, Eugene, OR, USA) and NanoDrop (Thermo Scientific, Waltham, MA, USA). The 16S rRNA genes were amplified using 16S V3-V4 primers: 16S amplicon PCR forward primer

5' TCGTCGGCAGCGTCAGATGTGTATAAGAGACAGCCTACGGGNGGCWGCAG

16S amplicon PCR reverse primer

5' GTCTCGTGGGCTCGGAGATGTGTATAAGAGACAGGACTACHVGGGTATCTAATCC

Input gDNA (12.5 ng) was amplified with the primers, and a subsequent limited‐cycle amplification step was performed to add multiplexing indices and Illumina sequencing adapters. The final products were normalized and pooled using PicoGreen, and the library size were verified using the LabChip GX HT DNA High Sensitivity Kit (PerkinElmer, Massachusetts, USA). The sequencing was performed using the MiSeq platform (Illumina, San Diego, CA, USA).

**2.2.5b: Next-generation sequencing and data analysis**. Next-generation sequencing (NGS) analysis was performed using the fecal samples by Macrogen Inc. (Seoul, Korea). The amplicons were sequenced using the 454 FLX titanium system. The paired-end reads obtained using NGS were assembled with FLASH software, and the sequencing reads were filtered and trimmed using the CD-HIT-OUT software and rDNA Tools. For the taxonomic analysis, operative taxonomic units (OTUs) were selected on the basis of 97% threshold of sequence similarity with the QIIME-UCLUST program. The filtered reads were clustered, and OTUs were generated using CD-HIT-DUP. The filtered sequences were analyzed using the QIIME pipeline, which includes features to calculate diversity indices and phylogenetic diversity (PD) rarefaction curves. The diversity indices (OTUs, Chao1, Shannon, and Simpson index) were measured. The Ribosomal Database Project classifier was used for taxonomic classification of the fecal microbiome of the control and treatment groups.

#### 2.2.6. Statistical analysis

The data were analyzed using the GLM procedure of SAS (SAS Institute Inc., Cary, NC, USA) in a randomized complete block design. The statistical model for growth performance and digestibility included effects of dietary treatment as fixed effects and initial BW as a covariate. Statistical significance and tendency were considered at *P* < 0.05 and 0.05 ≤ *P* < 0.10, respectively. The *in vitro* digestibility results were analyzed and compared using the *t*-test and SPSS software. A *P* value < 0.05 was considered statistically significant.

## Results

### 3.1. *In vitro* digestibility study

#### 3.1.1. IVID

The IVID of DM without multi-enzyme supplementation for corn meal, Oriental herbal extract, wheat meal, soybean meal, fish meal, IRG, and peanut hull was 95.24%, 85.91%, 83.02%, 76.12%, 67.19%, 31.6%, and 8.85%, respectively; IVID of DM with multi-enzyme supplementation was 97.66%, 87.62%, 85.76%, 76.29%, 66.56%, 31.44%, and 8.61%, respectively (Table 2). Among all the tested feed stuffs, corn meal showed a significant (*P* = 0.01) increase in DM digestibility with multi-enzyme supplementation. DM digestibility of wheat meal, soybean meal and Oriental herbal extract (*P* > 0.05) quantitatively increased with multi-enzyme supplementation (S1 Fig). However, IVID of DM for fish meal slightly (0.63%) decreased (*P* > 0.05). The *in vitro* ileal digestibility of peanut hull and IRG did not change with multi-enzyme supplementation.

**Table 2:** IVID of feed ingredients (values are mean ± standard deviation, n = 3).

Feed Ingredient	Control mean ± SD	Treatment mea ± SD	*P*-value
Wheat meal	83.02 ± 5.17	85.76 ± 2.41	0.452
Soybean meal	76.12 ± 0.51	76.29 ± 0.57	0.719
Fish meal	67.19 ± 1.09	66.56 ± 1.36	0.564
Oriental herbal extract	85.91 ± 0.54	87.62 ± 1.29	0.102
Peanut hull	8.85 ± 0.31	8.61 ± 0.51	0.529
Corn meal	95.23 ± 0.17	97.67 ± 0.27	0.001
IRG	31.6 ± 0.78	31.44 ± 0.45	0.769

SD, standard deviation; Control, without enzyme supplementation; Treatment, with enzyme supplementation IRG, Italian ryegrass

#### 3.1.2. IVTTD

The IVTTD of DM without multi-enzyme supplementation for Oriental herbal extract, soybean meal, corn meal, fish meal, wheat meal, IRG, and peanut hull was 95.54%, 90.64%, 88.91%, 85.67%, 84.33%, 33.6% and 11.8%, respectively; the IVTTD of DM with multi-enzyme supplementation was 96.84%, 91.32%, 97.61%, 84.93%, 87.47%, 33.14% and 9.6%, respectively (Table 3). On the basis of *in vitro* analysis, IVTTD of DM for corn meal (*P* = 0.0039) and Oriental herbal extract (*P* = 0.004) increased significantly (S2 Fig). However, the digestibility of peanut hull decreased (*P* = 0.032) with multi-enzyme supplementation. Among all the feed-stuffs, corn meal had the highest IVTTD, followed by Oriental herbal extract, and peanut hull which exhibited the lowest digestibility with multi-enzyme supplementation.

**Table 3:** IVTTD of feed ingredients (values are mean ± standard deviation, n = 3).

Feed Ingredient	Control mean ± SD	Treatment mean ± SD	*P*-value
Wheat meal	84.33 ± 3.05	87.47 ± 1.08	0.169
soybean meal	90.64 ± 0.37	91.32 ± 0.23	0.053
fish meal	85.67 ± 1.10	84.93 ± 0.85	0.411
Oriental herbal extract	95.54 ± 0.35	96.84 ± 0.12	0.004
peanut hull	11.8 ± 0.9	9.6 ± 0.76	0.032
Corn meal	88.92 ± 0.39	97.6 ± 0.18	0.003
IRG	33.6 ± 0.43	33.14 ± 0.72	0.395

SD, standard deviation; Control, without enzyme supplementation; Treatment, with enzyme supplementation IRG, Italian ryegrass

### 3.2. *In vivo* study

#### 3.2.1. Nutrient digestibility and apparent ileal and total tract digestibility

The apparent ileal and total tract digestibility of nutrients from control and treatment are provided in Table 4. Digestibility of DM, energy, and CP in pigs fed with control diet were not statistically different (*P* > 0.05) from that of pigs fed with treatment diet. Apparent ileal digestibility of DM in the control and treatment groups was similar (78.34% and 78.44%, respectively). The values of apparent ileal digestibility of energy in the control and treatment groups (71.86% and 72.14%, respectively) were similar to apparent ileal digestibility of CP (71.75% and 72.56%, respectively). The apparent total tract digestibility of DM, CP, and GE for control group was 80.45%, 80.76% and 74.76%, respectively, and 80.78%, 81.44%, and 75.46% for the treatment, respectively, and these values were not significantly (*P* > 0.05) different between the groups.

**Table 4:** Apparent digestibility of nutrients in pigs fed diets with multi-enzyme supplementation.

Item	Control^x^	Treatment^y^	SEM^z^	*P*-Value
Apparent ileal tract digestibility, %				
DM^a^	78.34	78.44	0.4	0.913
CP^b^	71.75	72.56	0.35	0.245
GE^c^	71.86	72.14	0.28	0.546
Apparent total tract digestibility, %				
DM	80.45	80.78	0.52	0.908
CP	80.76	81.44	1.45	0.678
GE	74.76	75.46	1.32	0.528

Control^x^; without multi-enzyme supplement

Treatment^y^; with multi-enzyme supplement

SEM^z^; standard error of mean

DM^a^; dry matter CP^b^; crude protein GE^c^; gross energy

#### 3.2.2. Growth performance

The average initial BW of piglets was 6.02 ± 0.32 kg and final BW of pigs in control and treatment group was 25 and 26.36 kg, respectively. Multi-enzyme supplementation had no significant effect on feed intake, ADFI, feed conversion efficiency, or growth performance of pigs (*P* > 0.05; Table 5).

**Table 5:** Growth performance of weaned pigs fed dietary treatment^1^.

Items	Control^x^	Treatment^y^	SEM^z^	*P*-value
**Phase 1 (d 1–21)**				
Initial BW, kg	6.02	6.03	0.42	0.975
Final BW, kg	13.41	14.09	0.54	0.714
Feed intake, kg	35.15	37.49	1.78	0.485
ADG, g/d	352	384	28.12	0.371
ADFI, g/d	558	595	22.29	0.456
G: F, g/g	0.631	0.645	0.034	0.514
**Phase 2 (d 21–42)**				
Initial BW, kg	13.41	14.09	0.54	0.714
Final BW, kg	25	26.36	1.54	0.465
Feed intake, kg	53.93	55.19	3.81	0.712
ADG, g/d	552	584	27.65	0.765
ADFI, g/d	856	876	34.75	0.647
G: F, g/g	0.645	0.667	0.032	0.698
**Overall (d 1–42)**				
Initial BW, kg	6.02	6.03	0.42	0.975
Final BW, kg	25	26.36	1.54	0.465
Feed intake, kg	89.08	92.67	4.45	0.624
ADG, g/d	452	484	28.91	0.601
ADFI, g/d	707	735.5	32.68	0.374
G: F, g/g	0.639	0.658	0.031	0.862

^1^Values are presented as the least squares mean of 6 replicates (3 pigs/replicate).

Control^x^ = diet based on corn and soybean meal.

Treatment^y^ = control with 0.1% multi-enzyme.

SEM^z^ = standard error of mean.

#### 3.2.3. Microbial community analysis

**Fig 2:**
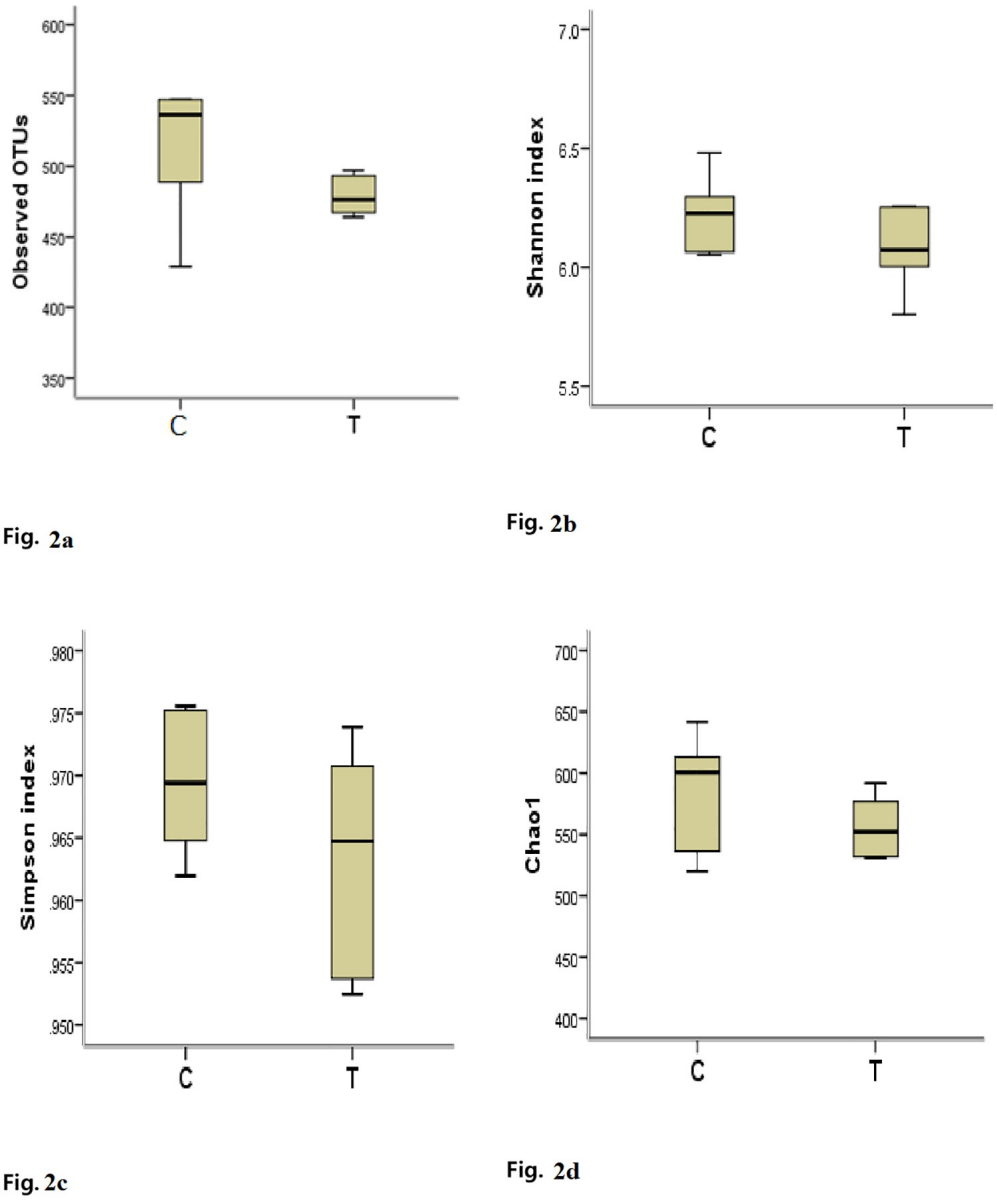
Variations in alpha diversity of pigs.

**Fig 3:**
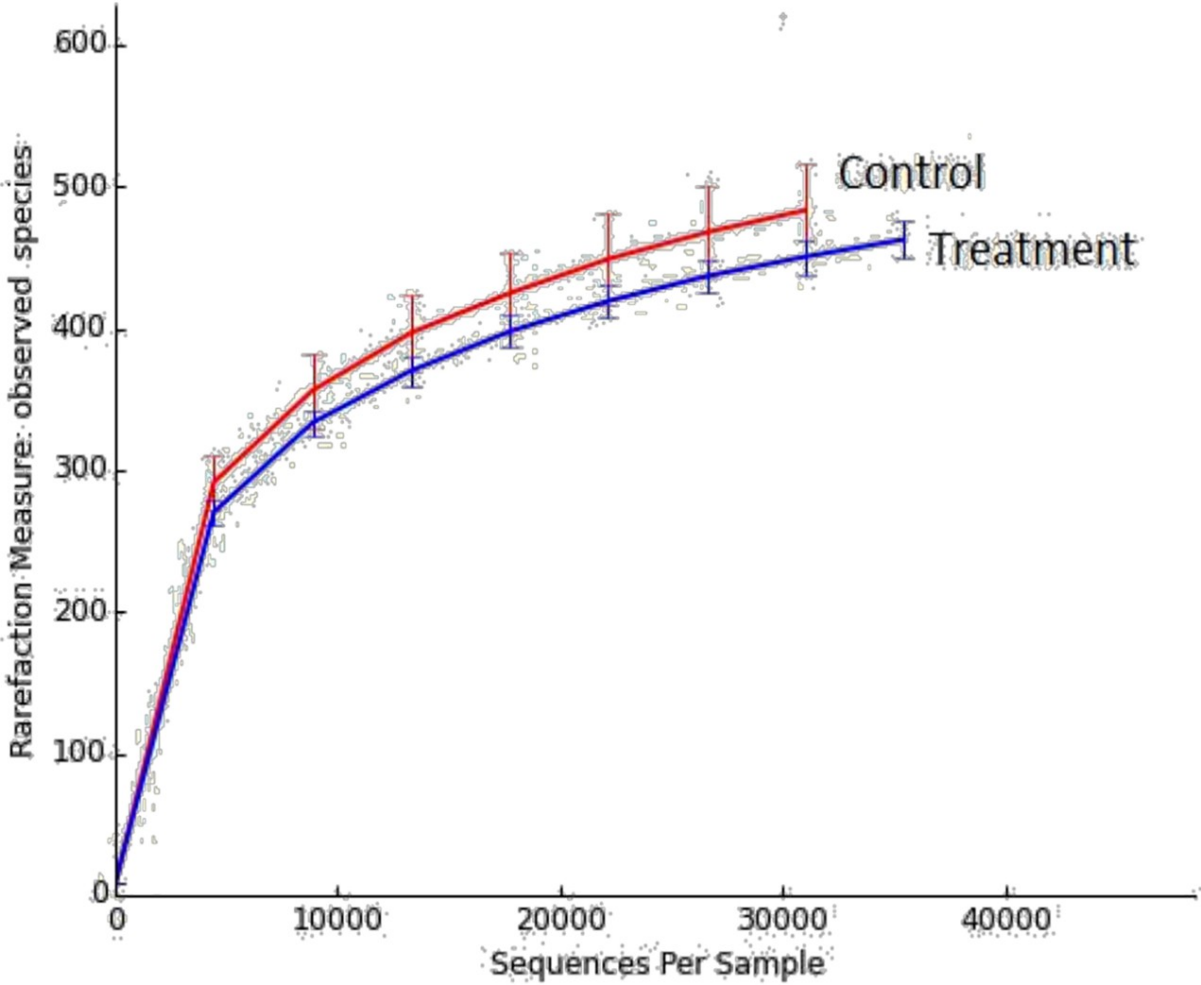
Rarefaction curves of observed species in groups. Control = without multi-enzyme; Treatment = with multi-enzyme.

**3.2.3a. DNA sequence data and bacterial diversity**: The pyrosequencing analyses generated a total of 523,130 valid sequences for the control and treatment groups by using the fecal samples of 12 pigs. The average OTUs at 97% confidence intervals were 514 OTUs for the control group and 479 OTUs for the treatment group (Fig 2a). Microbial diversity was calculated using the Shannon-Weaver and Simpson diversity indices (Fig 2b and 2c). The average Shannon-Weaver index of the control and treatment groups was 6.22 (SD = 0.16) and 6.07 (SD = 0.17), respectively. The Simpson index values of the control and treatment groups were 0.97 (SD = 0.005) and 0.96 (SD = 0.009), respectively. The alpha diversity measurement, chao1 estimator of total species richness value was 585 in the control and 556 in the treatment group, but not statistically significant (*P* = 0.208) differences were observed (Fig 2d). These results indicate that the treatment diet did not affect the diversity of microbiota in the gut (S1 Table). The total number of observed species in the control and treatment groups was presented using rarefaction curves (Fig 3).

**Fig 4:**
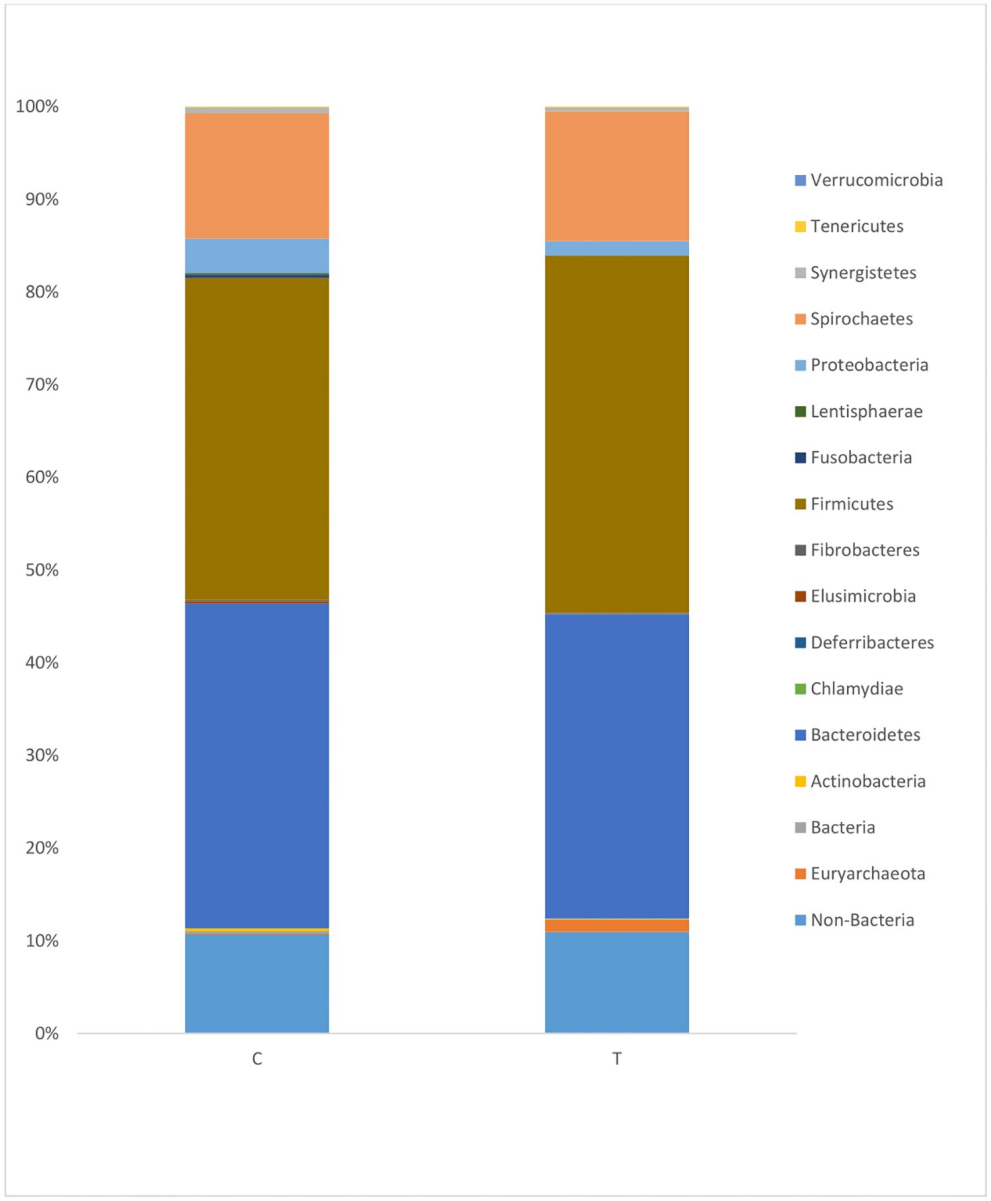
Bacterial taxonomic composition of phylum level.

**3.2.3b. Taxonomic analysis**: The results of the taxonomic analysis at the phylum level are shown in Fig 4. The bacteria in both control and treatment samples were predominantly belonged to Firmicutes and Bacterioidetes, which account for more than 65% of the total sequences. At the phylum level, the bacteria in the control group belonged primarily to the phyla Bacteroidetes (34.99%), Firmicutes (34.73%), Spirochaetes (13.50%), and Proteobacteria (3.72%); the other phyla and non-bacteria comprised 2.3% and 10.69%, respectively, of the total sequences analyzed. The bacteria in the treatment group primarily belonged to the phyla Firmicutes (38.52%), Bacteroidetes (32.82%), Spirochaetes (14.00%), Proteobacteria (1.55%) and Euryarchaeota (1.30%); the non-bacteria comprised 10.97% of the total analyzed sequences. Both groups shared similar phyla, with a trend toward higher abundance of Firmicutes and a corresponding decrease in the abundance of Bacteroidetes and Proteobacteria in the treatment group.

**Fig 5:**
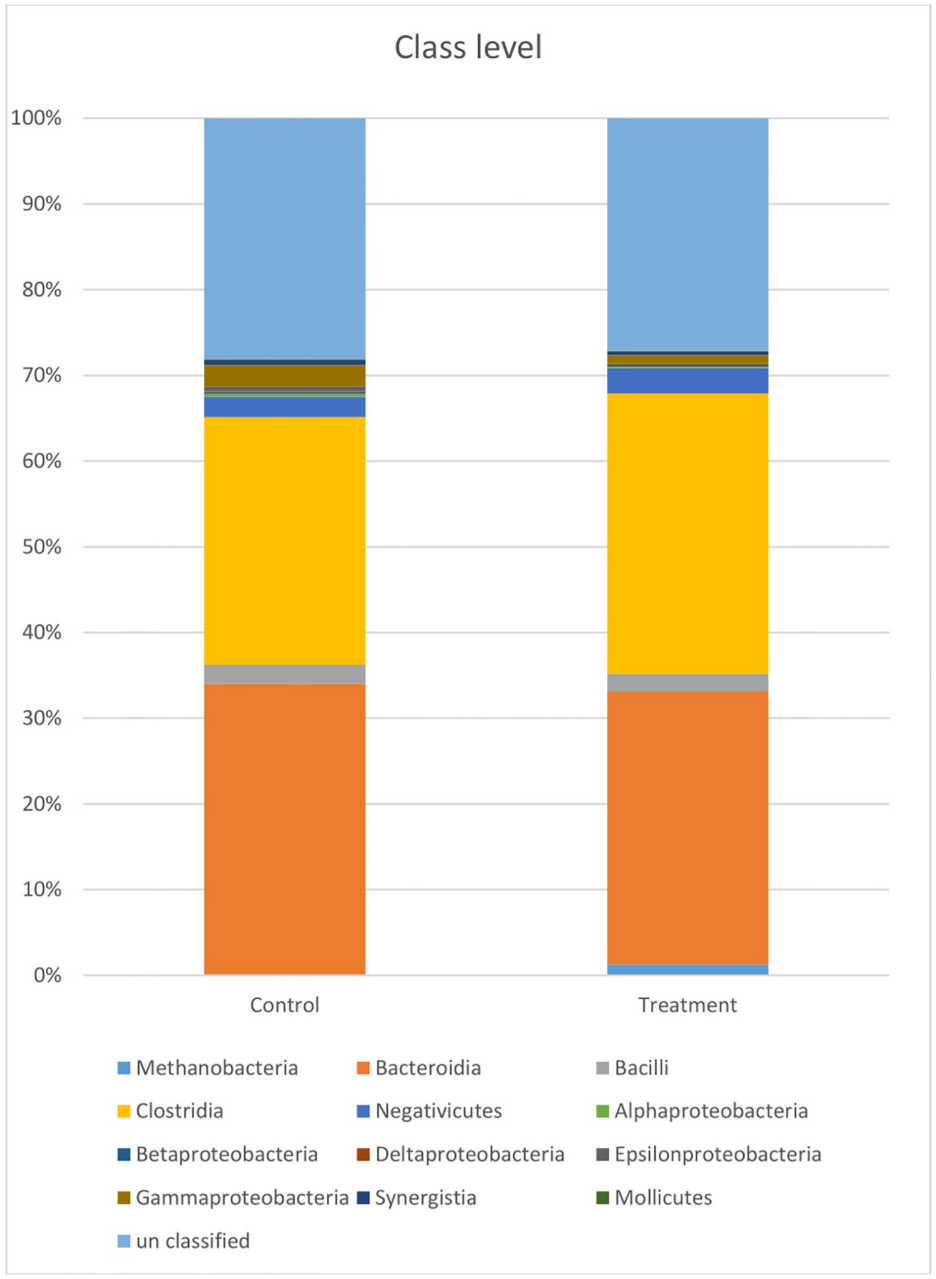
Bacterial taxonomic compositions of class level.

**Fig 6:**
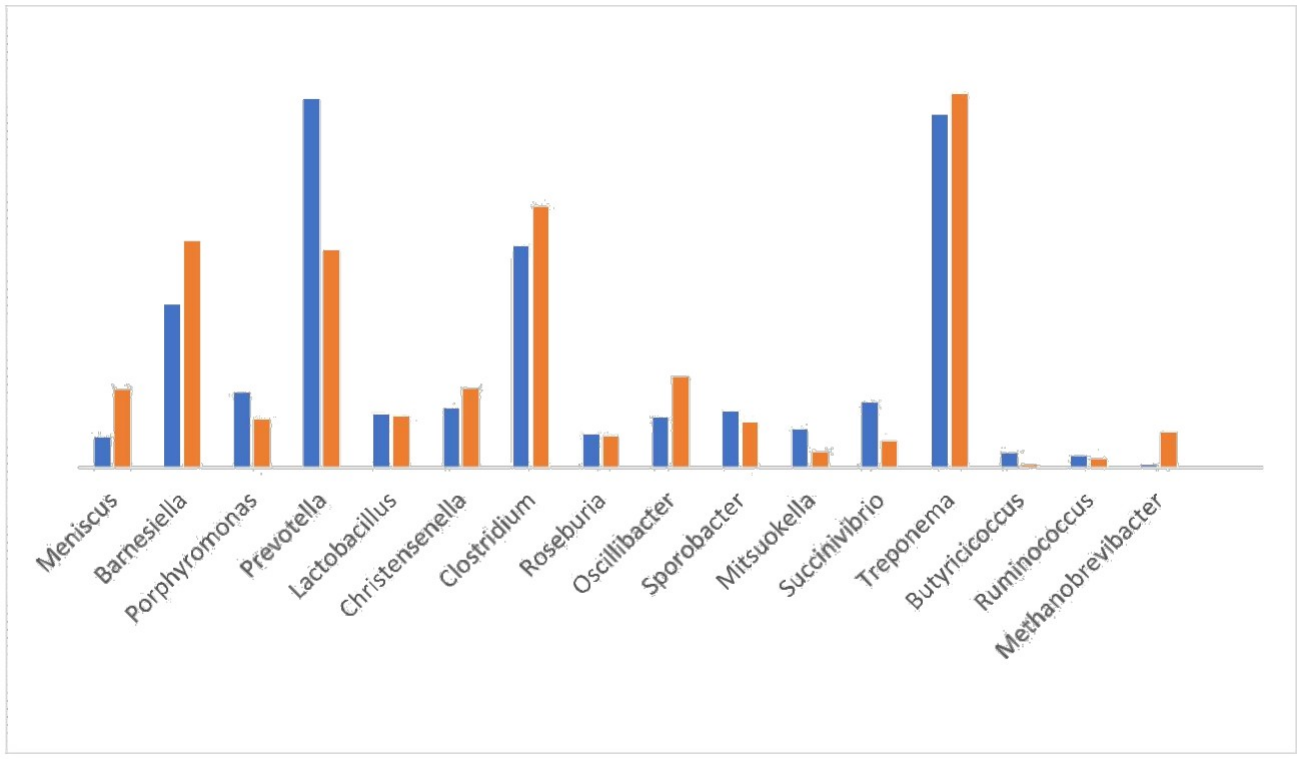
Bacterial taxonomic compositions of genus level.

At the class level (Fig 5), the proportion of Clostridia increased and the proportion of Bacteroidia and Gammaproteobacteria decreased in the treatment group. At the genus level (Fig 6), a total of 135 genera were identified. In the control group, more than 70% of the total sequences belonged to 13 genera: *Meniscus*, *Barnesiella*, *Porphyromonas*, *Prevotella*, *Lactobacillus*, *Christensenella*, *Clostridium*, *Roseburia*, *Oscillibacter*, *Sporobacter*, *Mitsuokella*, *Succinivibrio*, and *Treponema*. *Prevotella* (13.71%) and *Treponema* (13.13%) were the most abundant genera. A total of 124 genera were identified in the treatment group, and more than 65% of the total sequences belonged to 13 genera: *Methanobrevibacter*, *Meniscus*, *Barnesiella*, *Porphyromonas*, *Prevotella*, *Alistipes*, *Lactobacillus*, *Christensenella*, *Roseburia*, *Oscillibacter*, *Sporobacter*, *Phascolarctobacterium*, and *Treponema*. *Barnesiella*, *Prevotella*, and *Treponema* were the most abundant genera. However, the proportion of *Prevotella* decreased from 13.71% (control) to 8.06% in the treatment group, and the proportion of *Barnesiella* and *Treponema* slightly increased in the treatment group. The proportion of other genera, including *Meniscus*, *Butyricicoccus*, *Ruminococcus*, *and Succinivibrio*, decreased in the treatment group (S2 Table).

## Discussion

### 4.1. *In vitro* DM digestibility of feed-stuffs

*In vitro* ileal and total tract digestibility methods are effective techniques to evaluate the feed efficiency before using an animal diet. When compared with animal experiments, these techniques are cost–effective, rapid, and repeatable [24, 29]. In this study, we examined the DM digestibility of feed-stuffs that can be used as a basal diet (corn, fish, wheat, and soybean meal) and feed additives (Oriental herbal extract, IRG, and peanut hull). Numerous studies have reported the effects of exogenous supplementation of dietary enzymes (single enzyme/ multi-enzymes) on pig nutrition [7, 30]. In the present study, we used in vitro digestibility studies to evaluate feed stuffs with or without multi-enzyme supplementation. The multi-enzyme preparation mainly contained NSP-degrading enzymes (xylanase and glucanase). The purpose of this study was primarily to screen the digestibility of these feed ingredients for further animal experiments.

The multi-enzyme supplementation did not influence the IVID of DM digestibility for all the feed–stuffs, except corn meal. The corn meal ileal DM digestibility increased (*P* = 0.001) by 2% with enzyme addition. Park *et al*. [31] have reported similar findings. The results of IVTTD of DM digestibility for soybean, corn, and wheat meal were similar to those of previous studies [23, 31, 32]. The average IVTTD of DM digestibility for IRG (33.6%) was similar to that reported by Anderson and Ralston [33]. The DM digestibility of peanut hull decreased with multi-enzyme addition in this study. Similar results have been reported previously [34]. Because of a high proportion of crude fiber (60–67%) and only 6–7% crude protein, the digestibility of peanut hull is less than 20% in ruminants [35]. Lindermann *et al*. [36] studied the effects of peanut hull substitution (7.5, 15, and 30%) in swine diets. They reported no differences in daily gain among the treatments.

### 4.2. Effects of enzyme addition on the digestibility and growth performance of pigs

Corn meal and soybean meal are the main energy and protein sources in typical swine diets. All plant-based feed-stuffs have some ANF such as cellulose, gums, and hemicelluloses (arabinoxylans, mannan, and glucomannan). Corn contains 9.7% NSP, mainly 4.3% arabinoxylans and 0.21% phytate P [37, 38, 39]. Soybean meal has 21.7% NSP, mainly α-galactosides and β-galactomannan [40] and 0.38% phytate P [25]. However, NSP also has beneficial effects due to microbial fermentation of NSP in the hindgut [41]. The effects of NSP-degrading enzymes as feed additives for pigs have been reported in high fiber diets with wheat, rye, barley, and rice [30, 42]. However, only a few studies have investigated the effects of enzyme supplementation on corn-soybean meal diets [39, 43]. Therefore, the objective of this study was to investigate the effects of multi-enzyme supplementation of corn-soybean meal-based diets for pigs. Quantitatively, multi-enzyme supplementation increased (*P* > 0.05) the apparent total tract DM digestibility, energy, and CP digestibility. Jo *et al*. [39] have reported similar results. In contrast, Omogbenigun *et al*. [44] reported that the pigs fed enzyme-supplemented diets had higher (*P* = 0.001 to 0.014) total-tract digestibility of DM, CP, and GE than those fed the control diet. They used many feed stuffs as a basal diet, including corn and soybean meal.

The results of the growth performance experiment are consistent with those of previous studies [39, 45]. In the present study, the results of growth performance did not show significant (*P* > 0.05) differences among two groups. Similarly, Willami *et al*. [46] reported that enzyme supplementation to corn- soybean-based diets exerted no effect on the nutrient digestibility and growth performance of growing pigs. However, enzyme supplementation of high NSP containing wheat-rye barley-based diets showed positive effects on growth performance.

### 4.3. Effect of enzyme addition on the pig gut microbial diversity and community changes

The mammalian gut microbiota is a complex and diverse ecosystem composed of different microbial communities and there is evidence that the gut microbiota plays an important role in host health [47, 48]. The symbiotic relationship between the host and gut microbiota is well established [49, 50]. The composition of the microbiota can be affected by several factors such as age, diet, environment and host genetics. Few studies have examined the changes in microbial communities in response to various diets in pigs [51, 52]. In this study, we examined microbial community changes in young pigs fed a basal diet supplemented with multi-enzyme complex by using NGS. As shown in previous studies, gram-positive Firmicutes followed by gram-negative Bacterioidetes were the most dominant phyla in both groups [53]. The abundance of Firmicutes increased in the treatment group. The ratio of Firmicutes to Bacteroidetes shifts according to BW, and this proportion increases with weight gain [54, 55, 56]. The abundance of Proteobacteria, which includes various pathogen species, such as *Campylobacter* and *Succinivibrio*, declined with the enzyme supplementation. At the genus level, *Prevotella* and *Treponema* were dominant in both groups. *Prevotella* is mainly composed of gram-negative bacteria in the gastro-intestinal tract of pigs. The results were confirmed using previous culture [57] and DNA-based culture-independent studies [58, 59, 60, 61].

Dietary enzyme supplementation resulted in the higher populations of two genera, *Treponema* (Spirochaetes) with abundant *Treponema porcinum*, and *Barnesiella* (Bacteroidetes) with *Barnesiella intestinihominis*. In contrast, Zhang *et al*. [52] demonstrated that the abundance of *Treponema* decreased with enzyme supplementation in pigs fed wheat bran-based (WB) and soybean hull-based (SH) diets, and they observed that the proportion of *Treponema* was higher in the SH pigs than in the WB diet fed pigs. Furthermore, the treatment group showed decreased numbers of *Prevotella (Prevotella copri)*, *Clostridium*, *Butyricicoccus*, *Meniscus*, and *Succinivibrio*, which can act as pathogens and negatively affect the gut immunity. For instance, *Prevotella* species such as *Prevotella intermedia* and *Prevotella copri* can act as potential opportunistic pathogens [62, 63]. The Shannon index, which represents the species richness and evenness, and Simpson’s index, which accounts for proportional abundance or probability, were similar in both groups. Similarly, Zhang *et al*. [52] did not find significant differences in microbial diversity between two different fibrous diets with or without enzyme supplementation.

## Conclusions

Enzyme addition increased the *in vitro* total tract digestibility of Oriental herbal extract and corn meal. In the treatment group, multi-enzyme supplementation of the basal diet did not influence the nutrient digestibility and growth performance of the pigs. However, the microbial composition of the microbiota in the hindgut was modified by enzyme supplementation of the corn-soybean meal diet.

## Supporting information

S1 Fig *In vitro* ileal dry matter digestibility rates of feed stuffs without multi-enzyme (C) and with multi -enzyme (T). The results shown as mean ± standard deviation (*n* = 3). Significance measured at P < 0.05 and shown as asterisk (*).
(TIF)

S2 Fig *In vitro* total tract digestibility rates of feed stuffs without multi-enzyme (C) and with multi- enzyme (T). The results shown as mean ± standard deviation (*n* = 3). Significance measured at P < 0.05 and shown as asterisk (*).
(TIF)

S1 Table Community richness and diversity between control and treatment groups. (DOCX)

S2 Table Relative abundance of taxa in control and treatment groups representing > 0.1% of total sequences. (DOCX)
