# Supplementary figures and images for: Dietary multi-enzyme complex improves In Vitro nutrient digestibility and hind gut microbial fermentation of pigs
Source: PLoS One. 2019 May 28;14(5):e0217459. doi: 10.1371/journal.pone.0217459 (PMC6538249; doi:10.1371/journal.pone.0217459)

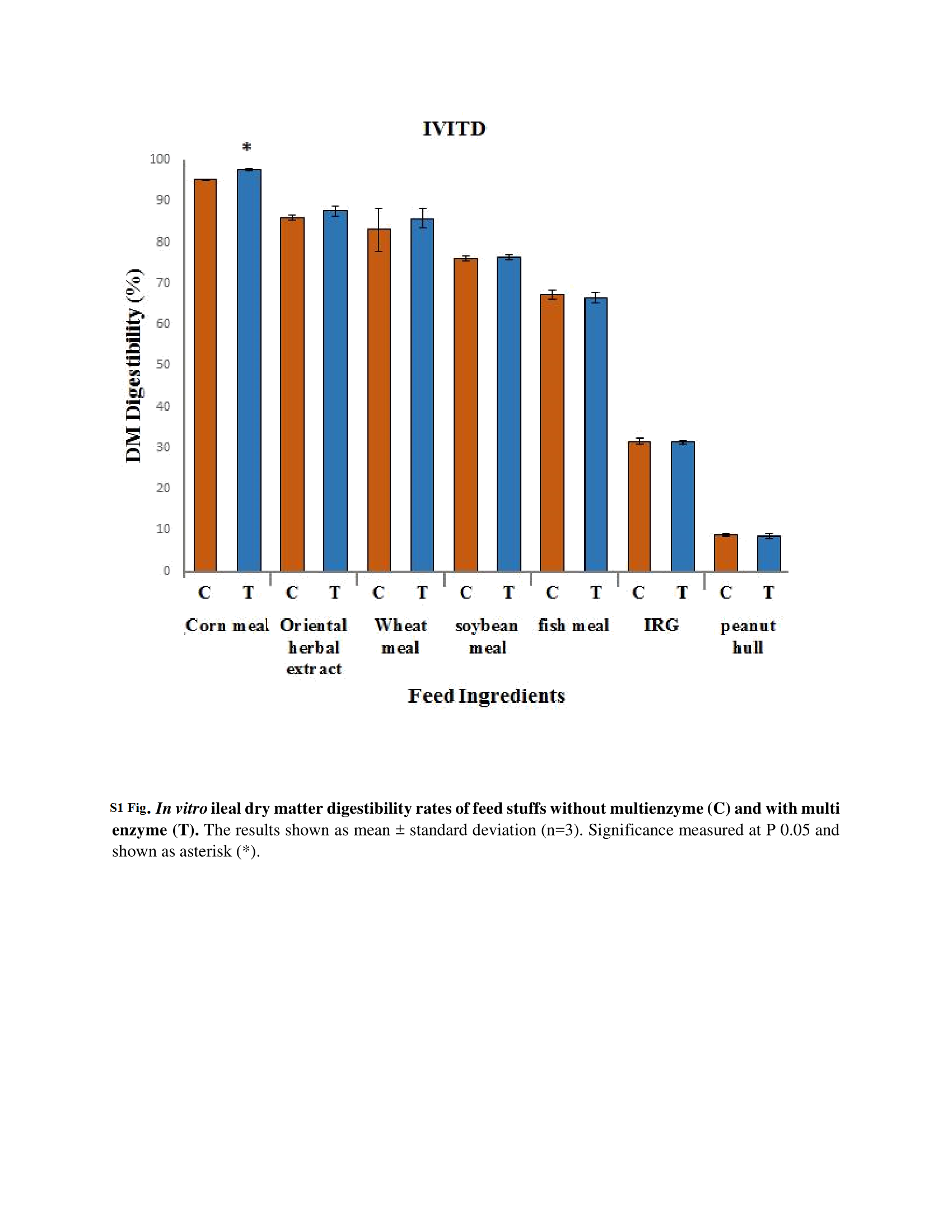

Supplement: S1 Fig — The results shown as mean ± standard deviation (n = 3). Significance measured at P < 0.05 and shown as asterisk (*). (TIF) [file pone.0217459.s001.tif]

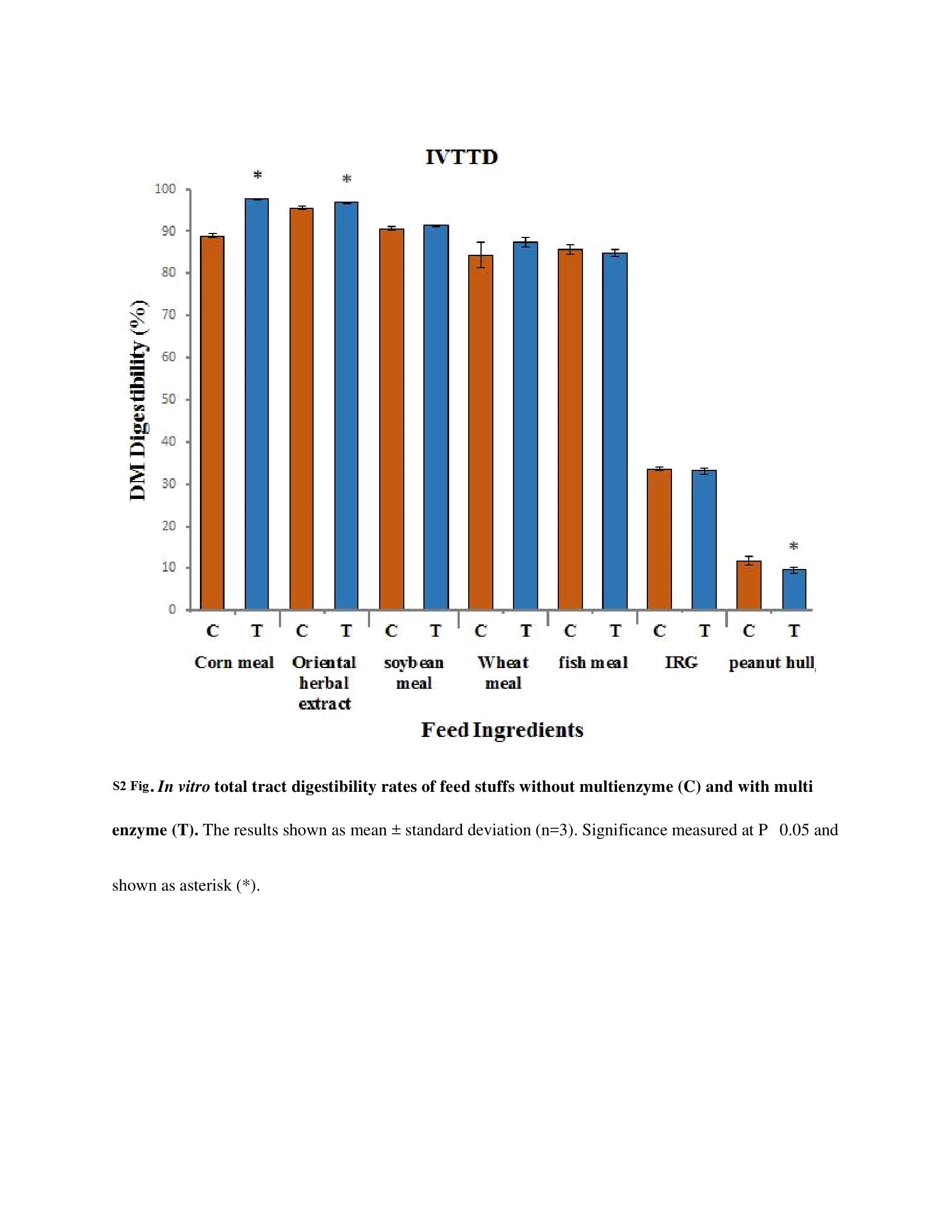

Supplement: S2 Fig — The results shown as mean ± standard deviation (n = 3). Significance measured at P < 0.05 and shown as asterisk (*). (TIF) [file pone.0217459.s002.tif]
