# Supplementary material for: Dietary multi-enzyme complex improves In Vitro nutrient digestibility and hind gut microbial fermentation of pigs
Source: PLoS One. 2019 May 28;14(5):e0217459. doi: 10.1371/journal.pone.0217459 (PMC6538249; doi:10.1371/journal.pone.0217459)
Supplement: S1 Table — (DOCX) [file pone.0217459.s003.docx]

**S1 Table. Community richness and diversity between control and treatment groups**

| Indices | Control Group Samples | | | | | | Treatment Group Samples | | | | | | P-Value |
| --- | --- | --- | --- | --- | --- | --- | --- | --- | --- | --- | --- | --- | --- |
|  | C1 | C2 | C3 | C4 | C5 | C6 | T1 | T2 | T3 | T4 | T5 | T6 |  |
| OUTs | 489 | 547 | 547 | 429 | 531 | 542 | 497 | 486 | 467 | 467 | 464 | 493 | 0.132 |
| Chao1 | 520 | 613.048 | 601.923 | 536.447 | 599.297 | 641.621 | 592.022 | 576.78 | 541.17 | 531.909 | 531.038 | 563.263 | 0.208 |
| Shannon index | 6.29467 | 6.28949 | 6.1642 | 6.05254 | 6.06381 | 6.48 | 6.00411 | 6.05224 | 6.09087 | 5.80038 | 6.25165 | 6.25511 | 0.154 |
| Simpson index | 0.97522 | 0.9705 | 0.96478 | 0.96197 | 0.96833 | 0.97559 | 0.95373 | 0.96457 | 0.96487 | 0.95245 | 0.97073 | 0.97389 | 0.183 |

Six samples for each group represented as C1-C6 and T1-T6
