# Supplementary material for: Dietary multi-enzyme complex improves In Vitro nutrient digestibility and hind gut microbial fermentation of pigs
Source: PLoS One. 2019 May 28;14(5):e0217459. doi: 10.1371/journal.pone.0217459 (PMC6538249; doi:10.1371/journal.pone.0217459)
Supplement: S2 Table — (DOCX) [file pone.0217459.s004.docx]

**S2 Table. Relative abundance of taxa in control and treatment groups representing > 0.1% of total sequences**

| Phylum | Classification | Control | | | | | | | Treatment | | | | | |
| --- | --- | --- | --- | --- | --- | --- | --- | --- | --- | --- | --- | --- | --- | --- |
|  |  | C1 | C2 | C3 | C4 | C5 | C6 | T1 | | T2 | T3 | T4 | T5 | T6 |
| Bacteroidetes | Prevotella | 21.02 | 19.76 | 6.33 | 12.36 | 9.35 | 12.86 | 12.43 | | 10.67 | 6.87 | 7.09 | 9.03 | 6.2 |
|  | Paraprevotella | 2.37 | 1.53 | 2.37 | 2.89 | 0.83 | 0.91 | 1.51 | | 1.52 | 2.01 | 0.77 | 4.03 | 2.13 |
|  | Barnesiella | 2.69 | 1.91 | 0.21 | 12.86 | 7.35 | 5.83 | 16.8 | | 1.89 | 3.29 | 14.25 | 10.85 | 3.4 |
|  | Porphyromonas | 2.01 | 0.76 | 7.66 | 0.96 | 1.41 | 2.25 | 0.71 | | 1.14 | 1.28 | 0.85 | 0.81 | 7.13 |
|  | Meniscus | 1.17 | 1.2 | 2.78 | 0.47 | 0.97 | 1.8 | 0.56 | | 8.93 | 4.47 | 2.02 | 0.33 | 0.97 |
|  | Parabacteroides | 0.26 | 0.32 | 1.99 | 0.5 | 0.24 | 0.93 | 0.68 | | 0.83 | 1.19 | 0.95 | 0.5 | 1.4 |
|  | Bacteroides | 0.23 | 1.2 | 0.74 | 0.72 | 0.44 | 0.86 | 0.27 | | 0.65 | 0.56 | 0.1 | 0.2 | 0.98 |
|  | Prevotellaceae | 0.46 | 1.17 | 0.61 | 1.51 | 0.49 | 0.56 | 0.35 | | 0.52 | 1.2 | 0.37 | 0.97 | 0.31 |
|  | Odoribacter | 0.01 | 0.01 | 0.08 | 0.09 | 0.08 | 0.06 | 0 | | 0.05 | 0.15 | 0.02 | 0 | 0.04 |
|  | Galbibacter | 1.69 | 0.97 | 0.68 | 1.12 | 0.38 | 0.52 | 0.48 | | 0.72 | 0.42 | 1.36 | 2.04 | 1.36 |
|  | Anaerophaga | 0.62 | 0.25 | 0.18 | 0.24 | 0.21 | 0.1 | 0.08 | | 0.1 | 0.17 | 0.29 | 0.05 | 0.03 |
|  | Bacteroidales | 0.85 | 8.92 | 1.97 | 7.23 | 8.41 | 5.17 | 1.3 | | 2.66 | 17.66 | 3.78 | 3.18 | 4.94 |
|  |  |  |  |  |  |  |  |  | |  |  |  |  |  |
| Firmicutes | Erysipelothrix | 0.52 | 0.35 | 1.57 | 0.21 | 0.25 | 0.28 | 0.26 | | 0.24 | 0.35 | 0.23 | 0.27 | 0.23 |
|  | Lactobacillus | 4.05 | 0.38 | 3.09 | 1.43 | 1.79 | 0.45 | 1.15 | | 0.73 | 1.59 | 2.7 | 3.32 | 2.36 |
|  | Clostridiales | 3.12 | 2.78 | 2.59 | 4.4 | 5.53 | 3.8 | 4.65 | | 2.31 | 3.29 | 4.51 | 7.37 | 7.4 |
|  | Clostridium | 6.57 | 5.29 | 8.98 | 13.8 | 9.7 | 6.96 | 5.85 | | 9.01 | 9.07 | 13.47 | 8.3 | 8.73 |
|  | Oscillibacter | 2.1 | 4.4 | 1.46 | 2.26 | 1.21 | 2.38 | 1.98 | | 2.4 | 3.34 | 4.65 | 3.2 | 4.29 |
|  | Roseburia | 1.51 | 1.7 | 0.55 | 1.17 | 1.26 | 1.87 | 0.35 | | 1.5 | 1.7 | 0.2 | 0.43 | 0.54 |
|  | Sporobacter | 1.54 | 1.4 | 1.13 | 0.64 | 0.83 | 0.64 | 0.45 | | 0.39 | 2.65 | 0.41 | 1.09 | 0.64 |
|  | Ruminococcus | 0.21 | 0.68 | 0.28 | 1.23 | 0.24 | 0.33 | 0.35 | | 0.26 | 0.07 | 0.18 | 0.64 | 0.19 |
|  | Peptococcus | 0.08 | 0.06 | 0.06 | 0.12 | 0.05 | 0.03 | 0.02 | | 0.04 | 0.04 | 0.03 | 0.12 | 0.01 |
|  | Peptostreptococcaceae | 4.52 | 2.47 | 2.6 | 3.85 | 2.02 | 1.05 | 1.58 | | 2.1 | 2.54 | 3.13 | 2.76 | 1.18 |
|  | Ruminococcaceae | 2.1 | 2.36 | 1.66 | 1.35 | 1.11 | 2.11 | 1.86 | | 1.23 | 0.7 | 1.04 | 1.28 | 3.68 |
|  | Acetanaerobacterium | 0.96 | 1.79 | 0.53 | 0.98 | 1.8 | 0.59 | 0.62 | | 0.6 | 0.45 | 0.7 | 1.13 | 3.26 |
|  | Mitsuokella | 0.33 | 0.61 | 0.23 | 2.49 | 0.4 | 0.31 | 1.67 | | 0.01 | 0 | 0.11 | 1.23 | 0.21 |
|  | Selenomonas | 0.26 | 0.58 | 0.1 | 0.81 | 0.25 | 0.07 | 0.62 | | 0.05 | 0.2 | 0.19 | 0.9 | 0.1 |
|  |  |  |  |  |  |  |  |  | |  |  |  |  |  |
| Actinobacteria | Streptomyces | 0.12 | 0.04 | 0.08 | 0.03 | 0.04 | 0.03 | 0.02 | | 0.02 | 0.05 | 0.05 | 0.07 | 0.27 |
|  | Actinomadura | 0.04 | 0.03 | 0.07 | 0.46 | 0.38 | 0.04 | 0.1 | | 0.27 | 0.21 | 0 | 0.57 | 0.3 |
|  |  |  |  |  |  |  |  |  | |  |  |  |  |  |
| Spirochaetes | Treponema | 10.4 | 12.2 | 14 | 7.82 | 16.62 | 13.01 | 11.6 | | 22.89 | 12.5 | 10.54 | 14.03 | 13.18 |
|  | Sphaerochaeta | 0.82 | 0.1 | 0.35 | 0.12 | 0.14 | 0.2 | 0.6 | | 0.16 | 0.08 | 0.04 | 0.07 | 0.12 |
|  |  |  |  |  |  |  |  |  | |  |  |  |  |  |
| Proteobacteria | Succinivibrio | 4.71 | 2.14 | 7.71 | 0.73 | 3.06 | 1.97 | 2.2 | | 1.38 | 0.8 | 1.1 | 0.64 | 0.27 |
|  | Helicobacter | 0.23 | 0.05 | 0.03 | 0 | 0.36 | 0.03 | 0.04 | | 0 | 0 | 0 | 0 | 0 |
|  | Campylobacter | 0.25 | 0.38 | 2.4 | 0.03 | 0.14 | 0.12 | 0.26 | | 0.31 | 0.01 | 0.13 | 0.2 | 0.25 |
|  | Desulfovibrio | 0.1 | 0 | 0.06 | 0.11 | 0.06 | 0.08 | 0.09 | | 0.08 | 0.15 | 0.13 | 0.21 | 0.1 |
|  | Marispirillum | 0.12 | 0.18 | 0.11 | 0.12 | 0.01 | 0.07 | 0.01 | | 0.01 | 0 | 0.02 | 0 | 0.01 |
|  |  |  |  |  |  |  |  |  | |  |  |  |  |  |
| Synergistetes | Dethiosulfovibrio | 0.57 | 0.36 | 0.7 | 0.35 | 0.65 | 0.39 | 0.7 | | 0.16 | 0.17 | 0.18 | 0.26 | 0.4 |
|  |  |  |  |  |  |  |  |  | |  |  |  |  |  |
| Lentisphaerae |  | 0.0054 | 0.03 | 0.21 | 0 | 0.06 | 0.79 | 0.25 | | 0.04 | 0 | 0 | 0 | 0 |
|  |  |  |  |  |  |  |  |  | |  |  |  |  |  |
| Planctomycetes | Rhodopirellula | 0.06 | 0.36 | 0.01 | 0.44 | 0.29 | 0.03 | 0.65 | | 0.04 | 0.03 | 1.03 | 0.11 | 0.17 |
|  |  |  |  |  |  |  |  |  | |  |  |  |  |  |
| Tenericutes | Acholeplasma | 0.11 | 0.08 | 0.06 | 0.02 | 0.15 | 0.01 | 0.09 | | 0.04 | 0 | 0.02 | 0.1 | 0.12 |
|  |  |  |  |  |  |  |  |  | |  |  |  |  |  |
| Archaea |  |  |  |  |  |  |  |  | |  |  |  |  |  |
| Euryarchaeota | Methanobrevibacter | 0.03 | 0.17 | 0.06 | 0.12 | 0.04 | 0.02 | 0.02 | | 0.03 | 2.76 | 6.14 | 0.62 | 0.3 |

Six samples for each group represented as C1-C6 and T1-T6
